# Stability of lithium treatment in bipolar disorder - long-term follow-up of 346 patients

**DOI:** 10.1186/2194-7511-1-11

**Published:** 2013-07-31

**Authors:** Anne Berghöfer, Martin Alda, Mazda Adli, Christopher Baethge, Michael Bauer, Tom Bschor, Paul Grof, Bruno Müller-Oerlinghausen, Janusz K Rybakowski, Alexandra Suwalska, Andrea Pfennig

**Affiliations:** Institute for Social Medicine, Epidemiology and Health Economics, Charité University Medical Center, Berlin, 10098 Germany; Department of Psychiatry, Dalhousie University, Halifax, Nova Scotia B3H 4R2 Canada; Department of Psychiatry and Psychotherapy, Charité University Medical Center, Berlin, 10098 Germany; Department of Psychiatry and Psychotherapy, University of Cologne Medical School, Cologne, 50923 Germany; Department of Psychiatry and Psychotherapy, Universitätsklinikum Carl Gustav Carus Dresden, Technische Universität Dresden, Dresden, 01307 Germany; Department of Psychiatry, Schlosspark Clinic, Berlin, 14059 Germany; Mood Disorders Center of Ottawa and Department of Psychiatry, University of Toronto, Toronto, M5S 2E4 Canada; Drug Commission of the German Medical Association, Berlin, 10623 Germany; Department of Adult Psychiatry, Poznan University of Medical Sciences, Poznan, 61-701 Poland

**Keywords:** Bipolar disorder, Lithium, Long-term treatment, Morbidity index, Stability

## Abstract

**Background:**

The purpose of this study was to investigate the effectiveness and stability of long-term lithium treatment in a prospective, international, multicenter cohort of bipolar patients in a naturalistic setting.

**Methods:**

Patients were selected according to DSM IV criteria for bipolar disorder and required long-term treatment. They were prospectively followed and documented in five centers belonging to the International Group for the Study of Lithium-Treated Patients. This was a prospective cohort study without a comparison group. Lithium treatment was administered in a naturalistic and specialized outpatient setting. All patients underwent a comprehensive psychiatric examination, which included the use of standard rating scales, as well as an evaluation of clinical course based on the morbidity index (MI).

Wald tests were used to assess the significance of fixed effects and covariates when analyzing the relationship between depressive, manic, and total morbidity index and several characteristics of illness course.

**Results and discussion:**

A total of 346 patients with bipolar disorder I or II were followed for a mean period of 10.0 years (standard deviation (SD) 6.2, range 1 to 20). The morbidity index remained stable over time: the mean MI was 0.125 (SD 0.299) in year 1 and 0.110 (SD 0.267) in year 20. The MI was not associated with the duration of lithium treatment, the number or frequency of episodes prior to treatment, or latency from the onset of bipolar disorder to the start of lithium treatment. The drop-out rate was high over the study period. Our findings suggest that long-term response to lithium maintenance treatment remains stable over time.

## Background

Lithium is recommended in all major international guidelines as a first-line prophylactic treatment for bipolar disorder (American Psychiatric Association 2002; Grunze et al. 2004; Crossley et al. 2006; Yatham et al. 2013; DGBS, DGPPN DGPPN 2012; Grof and Müller-Oerlinghausen 2009), and its efficacy in this context has been demonstrated in meta-analyses of controlled studies (Burgess et al. 2001; Geddes et al. 2004; Geddes and Goodwin 2006; Licht 2012). In naturalistic settings, however, the effectiveness of long-term lithium treatment has been reported to be much lower (Surtees and Barkley 1994; Harrow et al. 1990; Goldberg et al. 1995; Keller et al. 1993; Licht et al. 2008), and to even diminish over time (Post et al. 1993; Maj et al. 1989a). Maj and coworkers analyzed the course of illness in 43 bipolar patients who had been successfully treated with lithium for 2 years. During a follow-up period of 5 years, a substantial number of patients experienced recurrences despite their having been initially classified as responders to lithium treatment. The authors interpreted this as an indication that it may not be possible to achieve long-term stability with lithium prophylaxis (Maj et al. 1989a). Post and coworkers retrospectively assessed the course of illness in 66 lithium-refractory patients with affective disorder and found that a substantial number of those who initially showed a complete or partial response to lithium experienced a gradual loss of efficacy over time (Post et al. 1993). However, recent observational studies show a general superiority of lithium compared to alternative mood stabilizers in clinical practice (Kessing et al. 2012; Kessing et al. 2011; Nivoli et al. 2010; Garnham et al. 2007).

The differences observed in the degree of effectiveness across studies have been variously attributed to methodological disparities (Deshauer et al. 2005; Coryell 2009), a broadening of the diagnostic criteria for bipolar disorder (Grof et al. 1993; Grof et al. 1995; Grof 1998), or changes in the course of illness over long periods (Goodwin 1999).

The present study investigated a prospective, multicenter cohort of patients with bipolar disorder. Its aim was to determine whether the long-term effectiveness of lithium prophylaxis remains stable over time. A subgroup of 242 patients from this sample has been analyzed elsewhere with regard to the influence of atypical symptoms (Berghöfer et al. 2008). Another subgroup of 336 patients was included in an analysis of recurrence risk that applied extended Cox regression models, which allow for the use all follow-up data on diseases with multiple episodes, to examine the influence of atypical features on time to recurrence (Pfennig et al. 2010).

## Methods

### Inclusion criteria

Patients were selected based on classical criteria for diagnosing bipolar disorder. When the first patients were included in the study in the 1980s, the eighth revision of the International Classification of Diseases (ICD-8) (World Health Organization 1969) was in use. The ICD-8 was later replaced by the ICD-9 (World Health Organization 1979). After 1994, all of the patients in the study were rediagnosed according to DSM IV (American Psychiatric Association 1993). All patients required long-term treatment, as defined by the presence of at least one manic episode or at least two episodes of any type in the patient's history. Patients were included in the study if they had been treated continuously with lithium for at least 1 year and were at least 18 years of age.

From the time of their presentation at the clinic until 2004, all patients were followed up in the outpatient departments of five participating International Group for the Study of Lithium-Treated Patients (IGSLi) centers (Berlin, Germany; Halifax, Hamilton, and Ottawa, Canada; Poznan, Poland). These centers were founded in the 1980s and followed a standard research program consisting of long-term prophylactic treatment with lithium and other drugs for the management of unipolar mood disorder, bipolar mood disorder, or schizoaffective disorder (http://www.igsli.org; Müller-Oerlinghausen et al. 1994; Alda et al. 2000).

### Patient assessment

During each visit, patients were evaluated by a psychiatrist, who (a) performed a psychiatric assessment, taking account of the patient's case history and past medication; (b) administered one or more standard mood rating scales (Bech-Rafaelsen Melancholia and Mania Scales (Bech et al. 1979; Bech et al. 1978), Hamilton-Depression-Scale (HAMILTON 1960), Young Mania Scale (1978)); (c) performed a physical examination; (d) recorded any adverse events; and (e) prescribed any clinical or pharmacological interventions he or she felt was necessary. Serum lithium levels were also obtained. Patients averaged seven to eight visits each year, depending on comorbidity, severity of illness, and age. The number of visits per year was greater than in normal outpatient settings, facilitating optimal control of patients’ long-term prophylaxis. Psychiatric nurses and social workers were available to provide support during additional, unscheduled visits.

Before enrolling in the prospective cohort, patients were thoroughly informed about the study procedures, treatment, and possible side effects, and all participants gave written, informed consent. The study was approved by local research ethics committees in jurisdictions in which such approval was necessary. In the Poznan study center, the study was approved by the bioethics committee, Poznan University of Medical Sciences. The center in Ottawa had an umbrella approval from the Research ethics committee for the analysis of clinical data of lithium-treated patients (anonymously, with names removed). The center in Halifax had an approval from the ethics committee at Capital District Health Authority, Nova Scotia. In the Berlin center, an approval was not necessary because the subjects provided written informed consent to the anonymous and aggregate scientific use of data from their confidential medical records when admitted to the clinic.

The onset of bipolar disorder was defined as the first recorded diagnosis of bipolar disorder or, if this was lacking, as the first recorded symptoms clearly related to bipolar disorder. In turn, a recurrent episode was defined as the presence, in a previously remitted patient, of symptoms that required either psychotherapeutic or psychopharmacological treatment. All recurrences were recorded and graded in terms of severity, polarity, and duration. Finally, remission was defined as the absence of affective symptoms, as measured using standard mood rating scales. All data were collected prospectively.

First introduced by Coppen and Abou-Saleh (1982), the morbidity index (MI) was used in the present study as the outcome measure and includes severity and length of episodes. Severity is rated in a semiquantitative manner using three different degrees: symptoms that do not require treatment are rated as degree 1; symptoms that require psychotherapeutic or psychopharmacological treatment for acute affective illness but are manageable in an outpatient setting are rated as degree 2; and symptoms necessitating inpatient treatment for acute affective illness are rated as degree 3. We included symptoms of degrees 2 and 3 in the analysis and calculated the MI using the following formula:

For each year, the MI was calculated for all affective episodes (MI_total_) and also separately for depressive episodes (MI_dep_) and manic episodes (MI_man_). To remain in the study, patients were required to demonstrate sufficient compliance, which was defined as maintaining serum lithium levels of at least 0.5 mmol/L throughout the documentation period.

Antipsychotics, antidepressants, or anticonvulsants administered in addition to lithium were not regarded as prophylactic medication and were not included in the statistical analysis (a) if they were administered as part of maintenance treatment (i.e., in addition to lithium during the first 3 months after remission for the purpose of stabilizing the patient) or (b) if they were administered as acute treatment (i.e., in addition to lithium at any point 4 or more months after remission to manage new symptoms). In the latter case, weeks during which acute treatment with antipsychotics, antidepressants, or anticonvulsants was necessary were rated as degree 2 and were included in the morbidity index. In all other cases, treatment with drugs from any of these three drug categories was considered to be prophylactic in nature. Finally, other drugs, such as benzodiazepines, were not regarded as additional prophylactic medication, and no data on their use were recorded.

### Statistical analysis

Data were analyzed using BMDP (Biomedical Computer Programs) Statistical Software, Inc. (Cary, NC, USA), release 8.0. Unbalanced repeated measures regression models with structured covariance matrices were applied (module 5V in BMDP) to assess the impact of treatment duration on the yearly MI (within subject measure). Separate calculations were made for MI_total_, MI_dep_, and MI_man_. Maximum likelihood was used to estimate parameters, with the expected response values being expressed as a linear function of the parameters. The main advantage of this approach was that all subjects could be included regardless of their duration of treatment. Model selection was based on optimization of Akaike's information criterion (AIC) (Akaike 1973). The significance of the independent variables was estimated using the Wald test. A 5% level of significance was established with two-tailed tests. Using the same method, it was possible to examine the impact of the number of episodes before the start of lithium treatment, as well as of treatment delay, on the MI. Finally, independent variables were modeled in this analysis as covariates.

## Results

**Figure 1:**
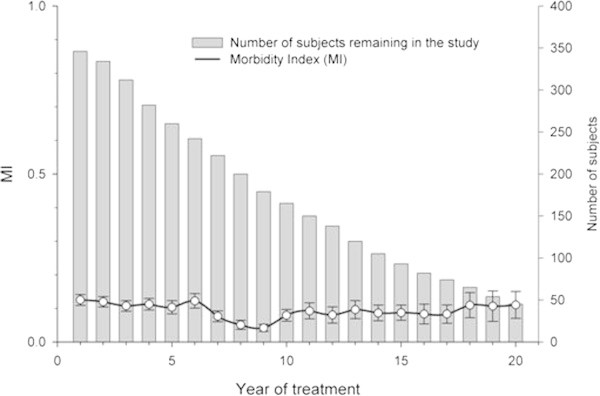
**Morbidity index over 20-year observation period.** Black lines show mean (SD) morbidity index for all affective episodes (MI_total_); gray bars show number of subjects in the analysis contributing to the morbidity index each year.

In the present study, a total of 346 patients were followed up for a mean period of 10 years (range, 1 to 20 years) on lithium treatment (Figure 1). The number of subjects varied between the participating treatment centers (Berlin *n* = 151, Halifax *n* = 35, Hamilton *n* = 14, Ottawa *n* = 75, and Poznan *n* = 71). Patients' baseline characteristics are given in Table 1. The mean age at the onset of bipolar disorder was 29, and lithium treatment was initiated with a mean latency of 10 years. For all 346 patients, the mean MI_total_ decreased slightly (i.e., from 0.125 to 0.110) over the 20-year observation period.

**Table 1:** **Baseline characteristics of the 346 study subjects**

Men, *n* (%)	147 (42.5)
Women, *n* (%)	199 (57.5)
DSM IV diagnosis, *n* (%)	
Bipolar I	270 (78.0)
Bipolar II	76 (22.0)
Length of follow-up period (year, SD, range)	10.0 (6.2, 1 to 20)
Age at onset of bipolar disorder (year, SD, range)	29.2 (11.0, 11 to 66)
Latency before start of lithium treatment (year, SD, range)	9.7 (9.3, 0 to 44)
Number of episodes before start of lithium treatment (*n*, SD, range)	5.5 (4.9, 0 to 40)
Comedication in all 346 patients (mean number of weeks [per follow-up year] during which any drugs from three different drug categories were administered; *n*, SD, range)	
Total	9.8 (17.7, 0 to 91)
Antipsychotics	2.5 (7.9, 0 to 52)
Antidepressants	3.5 (9.4, 0 to 52)
Anticonvulsants	3.8 (10.5, 0 to 52)
Comedication in 152 patients without lithium monotherapy (mean number of weeks [per follow-up year] during which any drugs from three different drug categories were administered; *n*, SD, range)	
Total	22.4 (20.9, 0 to 91)
Antipsychotics	5.6 (11.1, 0 to 52)
Antidepressants	8.0 (12.9, 0 to 52)
Anticonvulsants	8.8 (14.4, 0 to 52)

For long-term stabilization, a total of 152 patients received concomitant treatment with antidepressants, antipsychotics, or anticonvulsants. The mean period of concomitant treatment with a drug from one of these three categories was 22.4 weeks per year (Table 1). Altogether, 194 patients remained on lithium monotherapy for their entire follow-up period.

The results of the repeated measures regression did not show a change in the MI_total_, MI_dep_, or MI_man_ in the course of the study (see Table 2). There were also no significant associations between the number of episodes before the start of lithium treatment, the latency between the onset of illness onset and the start of lithium treatment, and the MI_total_, MI_dep_, or MI_man_ (see Table 2).

**Table 2:** **Relationship between depressive, manic, and total morbidity index and several characteristics of illness course (Wald tests for significance of fixed effects and covariates)**

Parameter	Chi-square	***P***
Total morbidity index		
Year of treatment; df = 19	19.847	0.404
Year of treatment × group membership interaction; df = 19	17.658	0.545
Number of episodes before index; df = 1	0.602	0.438
Number of recurrences before index; df = 1	0.002	0.963
Latency between onset of illness and start of lithium treatment; df = 1	1.991	0.158
Depressive morbidity index		
Year of treatment; df = 19	26.112	0.127
Year of treatment × group membership interaction; df = 19	12.208	0.877
Number of episodes before index; df = 1	0.304	0.581
Number of recurrences before index; df = 1	0.773	0.379
Latency between onset of illness and start of lithium treatment; df = 1	2.778	0.096
Manic morbidity index		
Year of treatment; df = 19	22.963	0.239
Year of treatment × group membership interaction; df = 19	16.271	0.639
Number of episodes before index; df = 1	0.305	0.581
Number of recurrences before index; df =1	2.105	0.147
Latency between onset of illness and start of lithium treatment; df = 1	0.013	0.908

Many patients dropped out of the study during the follow-up period. In total, 165 subjects were observed for at least 10 years, 93 for at least 15 years, and 45 for at least 20 years. Patients left the study for one of four reasons: (1) they had been in treatment for less than 20 years by the end of the study; (2) lithium side effects or interactions with a drug prescribed for a somatic comorbidity caused them to switch to another long-term prophylactic agent; (3) they switched to another outpatient clinic or moved and were lost to follow-up; or (4) they died. In patients who left the study, the MIs for the year of drop-out were not higher than the mean MI from the preceding years (*t* test, all *P* values = n.s.).

## Discussion

In the present study, the MIs remained stable throughout the observation period, confirming that the course of illness also remained stable over time in this subgroup of bipolar patients receiving prophylactic lithium treatment. No association could be found between the MIs and the number of episodes before the start of lithium treatment or the latency between the onset of illness and the start of lithium treatment.

In addition to this main finding, our study differs in several respects to investigations that have demonstrated poor stability with long-term lithium treatment. Many of the previous prospective studies on lithium treatment have had relatively short observation periods (i.e., less than 2 years in duration). Indeed, only a few have had longer observation periods, i.e., extending up to 5 years (Maj et al. 1989b; Maj et al. 1998; Maj 2003) or 7 years (Vestergaard and Schou 1988). In the present study, however, data were collected over a much longer period, covering up to 20 years.

Although other studies have assessed large cohorts over long observation periods, they have not focused on the long-term stability of prophylactic lithium treatment. For example, the mood disorders center in Sardinia, a Stanley Foundation Bipolar Network research center (Post et al. 2001; Suppes et al. 2001), evaluated a large cohort of lithium patients comparable in size to the IGSLI cohort. Tondo and coworkers presented comprehensive data on the long-term course of their lithium-treated patients within the Sardinian cohort (Tondo et al. 1998; Baldessarini et al. 2000). The results of an analysis over a mean treatment period of 6 years show a substantial improvement in the course of illness during long-term lithium treatment compared to the period before lithium treatment was initiated, although complete protection against affective episodes was uncommon. However, the issue of stability over time in patients on long-term lithium treatment was not addressed in this analysis (Tondo et al. 2001). Rybakowski and coworkers analyzed the efficacy of long-term lithium treatment, comparing the pre-index period with a post-index lithium treatment period of 10 years (Rybakowski et al. 2001). The study examined whether the effectiveness of lithium treatment in patients who initiated treatment in the 1980s was lower than that observed in patients who initiated treatment in the 1970s. Although patients in the 1970s group were maintained on higher serum lithium levels, no decrease in the effectiveness of treatment was observed in the 1980s group.

Several studies have evaluated long-term outcomes in patients who began with lithium treatment but continued with various treatments other than lithium in the naturalistic setting. A recent study by Licht and coworkers found an unsatisfactory outcome after 15 years (Licht et al. 2008); however, their follow-up was based on registry data that did not contain information on whether patients had continued to receive lithium. As a result, their findings do not allow inferences on the effectiveness of long-term lithium treatment.

Our study was not concerned with the efficacy or effectiveness of lithium, both of which have been demonstrated in a substantial body of literature. The use of the MI as our outcome measure did not allow us to compare the pre-index and post-index course of illness, because the MI requires prospective follow-up to obtain valid results. Retrospective data (e.g., from patient histories) are insufficient in this regard.

The present study also differs from other investigations regarding the indication for starting lithium prophylaxis. Most studies which have been performed during the last decade included patients within a broader definition of bipolar disorder and patients with an episodic pattern of illness are systematically underrepresented (Coryell 2009; Grof et al. 1995; Goodwin 1999; Gershon et al. 2009). The lithium clinics involved in this study, however, follow the Kraepelinian tradition of diagnosing bipolar disorder. As a result, it is conceivable that most of the patients in our sample were bipolar in the traditional and narrow sense of the term.

In addition, many of the newer studies perform analyses that use time-to-new-episode or time-to-new-rehospitalization or hazard ratios for relapse as the main outcome measure for long-term prophylactic effectiveness (Bowden et al. 2003; Tohen et al. 2005; Viguera et al. 2001; Geddes et al. 2010; Suppes et al. 2009). Although this type of analysis is well suited to relatively short trials that aim at proving a single drug's efficacy, it is inappropriate for long-term maintenance studies because it fails to discriminate between different types of response. Outcome criteria such as relapse or recurrence do not afford proper assessment of the course of illness in patients who show substantial clinical improvement but still experience episodes and thus fail to consider a patient-focused perspective which is relevant for clinical practice (Murru et al. 2011). Given that bipolar disorder is characterized by wide variations in the length and severity of episodes, the MI is an outcome measure which allows different forms of response and clinical course to be distinguished from one another in a precise fashion. This can be seen in an investigation of lithium maintenance treatment over a maximum of 15 years in a small subsample of the population in the present study: although the MI_total_ remained stable throughout the study period, the analysis of the absolute number of recurrences failed to produce any conclusive results because of the general shift over the study period from outpatient to inpatient treatment (Berghöfer et al. 1996). The outcome measure ‘burden of illness’ which is comparable to the MI and uses a life chart method combining severity and duration of episodes has recently been presented by Backlund and coworkers in a long-term evaluation (Backlund et al. 2009 ).

In summary, the MI appears to be the most accurate approach to describing chronic illnesses and would therefore seem to be a much more appropriate tool than survival analysis. Extended Cox regression models can provide a more accurate description of chronic illnesses because they focus on multiple recurrences rather than time to first recurrence (Pfennig et al. 2010).

The results of the present analysis are in agreement with those of several studies from the same group of researchers and, in part, derived from the same patient data. Berghöfer et al. used the MI to report on long-term response in a subgroup of bipolar patients over a maximum of 15 years (Berghöfer et al. 1996), as noted above, and in another study over a maximum of 20 years (Berghöfer and Müller-Oerlinghausen 2000). In both studies, which included a subset of subjects from the present investigation, the severity and duration of recurrences remained stable, and even decreased, over the observation period, albeit in small sample sizes. Two recent reviews also support our finding that the effectiveness of lithium prophylaxis does not diminish over time (Burgess et al. 2001;Kleindienst et al. 1999).

There has been some controversy as to whether the length of time between illness onset and the start of prophylactic treatment (i.e., latency) may influence patients' response to long-term treatment (Franchini et al. 1999). For this reason, we included latency of prophylactic treatment in our analysis. However, like the present analysis, other recent studies have not shown any association between negative outcomes and latency (Baethge et al. 2003a; Baethge et al. 2003b; Baldessarini et al. 2003).

Our study has several methodological limitations. Firstly, the severity of episodes may have been rated differently at the various centers due to the use of different symptom thresholds for the initiation of treatment. This clearly has the potential to affect which symptoms were rated as degree 2. In addition, with multiple countries and cultures involved, treatment selection may have varied depending on factors such as the healthcare system, the regional facilities available, and individual patient preferences. As in any long-term investigation, patients who receive up to 20 years of treatment were seen by a large number of therapists with varying degrees of training. However, the influence of the abovementioned factors may have been mitigated by the similar tradition of diagnosis and treatment followed by all of the centers that participated in the present study. More specifically, the centers agreed on a common treatment concept that gives preference to lithium monotherapy whenever possible as a means to avoid adverse events and drug-induced cycling. In addition, there were no differences in the MI between the centers. As a result, any center-specific effect is likely to have been relatively small.

Secondly, the centers participating in the present study were specialized academic outpatient clinics that, for the most part, treated patients who required an above-average amount of care. As such, a selection bias must be assumed. It should be noted, however, that the use of additional medication in our sample was quite low. Out of 346 patients 152 (44%) had a mean co-medication period of 22.4 out of 52 weeks (see Table 2), which indicates that patients with a severe course of illness were unlikely to have been overrepresented. The use of co-medication was higher in other long-term observations (e.g., 15). Because the present study is not an epidemiological investigation with a representative sample of bipolar patients, our results cannot be extrapolated to the general population of these patients; similarly, it is not possible to fully apply our results to routine psychiatric practice.

Thirdly, this analysis did not count affective symptoms that had been rated as degree 1 (i.e., symptoms that do not require additional treatment). Recently, a substantial number of studies have been conducted to assess interepisodic subthreshold symptoms, such as cognitive or affective impairment. It seems unlikely, however, that including degree 1 symptoms in the analysis would significantly affect the long-term stability shown by the MI.

Fourthly, a substantial number of patients dropped out of the study before completing 20 years of treatment, and these subjects were not followed up. One might argue that analyzing only those patients who remained on lithium treatment caused a selection toward higher stability, because non-responders may have switched to a different long-term medication or treatment setting. However, it should be noted that the mean MI in patients who dropped out was not higher during their last year of follow-up than it had been during the preceding years. This indicates that the course of illness in subjects who left the study was no worse than those who continued lithium treatment.

As a final consideration, it should be pointed out that the MI does not fully reflect the effects and benefits of lithium in individual patients. A patient might show a higher MI than another patient during lithium treatment but might nevertheless experience a substantially greater reduction in his or her affective morbidity after starting treatment. For example, a patient with an MI of 0.125 may have spent 15 days in the hospital (degree 3) and may show no other illness burden during a period of 1 year, alternatively the patient could have received approximately 23 days of treatment in addition to lithium for any affective symptoms without having spent any time in the hospital (degree 2). To show individual benefits, data comparing pre- and post-treatment MI would have been helpful. However, assessing the initial effectiveness of lithium treatment was not the primary focus of our analysis.

## Conclusions

Our results show that patients who met both the classical ICD-8 and ICD-9 criteria, as well as the DSM IV criteria, for bipolar disorder had a stable course of illness during long-term lithium treatment.

## Authors’ information

All authors are members of the International Group for the Study of Lithium Treated Patients (IGSLi, http://www.igsli.org).
